# Student perceptions of handover diaries and reflective learning in an undergraduate MBChB anatomy course.

**DOI:** 10.12688/mep.19946.1

**Published:** 2023-12-05

**Authors:** Seaneen McDougall

**Affiliations:** 1 Centre for Anatomy and Human Identification, University of Dundee, Dundee, Scotland, DD1 5EH, UK

**Keywords:** MBChB, anatomy, reflection, learning, dissection, diary

## Abstract

**Background:**

The time spent on basic sciences, including clinical anatomy, is decreasing in many medical curricula. While dissection is often seen as a cornerstone of medical education, there is increasing pressure to ensure time spent undertaking dissection is an efficient use of student time. As part of an MBChB clinical anatomy course , 1 ^st^ year students were asked to complete ‘dissection handover diaries’, designed through evidence-based pedagogy on reflection and engagement, which encouraged them to reflect on the dissection session and consider clinical applications of the anatomy covered in the session. Student engagement with an activity is important for it to be beneficial to their educational experience. This engagement is often increased when students perceive the activity to be useful to their learning.

**Methods:**

A survey was conducted, over two practical lab sessions on the final day of year one dissection in March 2023, using five Likert-type questions and one free-text question to evaluate student perceptions of the newly introduced dissection handover diaries. The survey was developed based on similar studies investigating student preference in dissection-based activities and questions were designed to elicit student perceptions on the usefulness of the diaries with respect to encouraging reflection, clinical application and student engagement in the sessions. Students were asked for constructive comments about the diaries in a free-text response question. Analysis was conducted using quantitative frequency distributions of survey responses as well as qualitative thematic analysis of the free text question.

**Results:**

Of a total of 228 students, 64 participated in the survey, a response rate of 28%. The results were positive overall, with many respondents identifying the diaries as beneficial for reflection, consolidation, clinical application, and engagement.

**Conclusions:**

Students perceived the dissection handover diaries to be useful to their anatomical learning, as well as encouraging reflection and application of knowledge.

## Introduction

The time spent on basic sciences, including clinical anatomy, is decreasing in many medical curricula ( Smith *et al.*, 2022). In addition, the approach to medical education is evolving in response to the requirement to fulfill medical competencies as required by the General Medical Council UK and the Medical Licensing Assessment ( Chew *et al.*, 2021; Sharma *et al.*, 2019) . To maintain anatomical knowledge, understanding and application at an acceptable level despite these challenges, many anatomy departments are investigating ways in which to ensure the time students spend in anatomy sessions is maximally effective ( Castellano *et al.*, 2023; Iwanaga *et al.*, 2021; Uruthiralingam & Rea, 2020). Dissection has long been known as a cornerstone of anatomical medical education and those departments which continue to practice it face pressure to ensure it is the most efficient use of student time in a busy curricula ( Jeyakumar *et al.*, 2020). Enabling students to make the most of their time in dissection classes can be achieved by using adjunct activities to encourage synthesis of learning ( Holland & Pawlikowska, 2019; Johnson *et al.*, 2012).

Reflection has been demonstrated to foster a deeper engagement and understanding of learning concepts as it encourages critical thinking in students ( Ashwin *et al.*, 2020), with improvements in their performance being noted as a result of the reflection upon the activities they have undertaken ( O’Loughlin & Griffith, 2020). As described by Sandars (2009), the metacognitive process of reflection allows students to use the learning experiences that they have already had to guide their next learning experiences – and in doing so the depth of their understanding improves. Reflective practice and its relationship to critical thinking was first discussed by Dewey (1933) as a process by which students could begin to make sense of experiences they found challenging. The suggestions he posed included event recall, explorative questions about why events occurred as they did and thinking about what actions would be required for a different outcome. This was further developed by the Experiential Learning Theory ( Kolb, 1984) in which the learning experience is described as being transformed by a cycle of experiencing, reflection, thinking and action – leading to a deeper understandings and better appreciation of the learning material.

In the context of medical education, reflective practice is increasingly being seen as a way in which to improve the development of professional practice ( Mamede & Schmidt, 2004). The importance of this is emphasised by reflective practice being considered a core component of medical student outcomes by the General Medical Council (GMC) in the UK ( General Medical Council, 2022). Anatomical education offers many opportunities for reflective learning ( Lachman & Pawlina, 2006) including self-directed learning in dissection, critical-thinking assessment approaches and ‘meet the cadaver’ orientation, amongst others. Some educators have taken advantage of this opportunity and have implemented reflective practice approaches in the anatomy curriculum, particularly within the dissection course ( Alt-Epping *et al.*, 2014; Anstey *et al.*, 2014; Shiozawa *et al.*, 2020). Where implemented, these reflective practice inclusions have been reported as being successful in contributing to the development of professional identity in the medical students taking part, as well as allowing students to consider the context of death and dying more deeply. Despite this, the use of reflective practice in anatomical education is reported as being relatively understudied ( Abrams *et al.*, 2021).

In addition to reflective practice being important for professional identity, it is thought that reflection on understanding of subject matter may aid students in deeper understanding of the concepts being taught. A study by O’Loughlin and Griffith (2020), demonstrated that undergraduate anatomy students who participated in a reflective practice process through journalling, reported feeling more confident in their understanding of the concepts, as well as improving their metacognitive skills. The same study also discussed the evidence produced during the reflective exercise showing how students become ‘expert learners’ in the subject matter, in this case application of anatomical knowledge to medical imagery. This indicates that it may be possible to use reflective practice to aid medical student understanding of anatomy and its application to clinical scenarios.

Learning and application of knowledge has been shown to be more effective when students are actively engaged in their learning. In turn, engagement has been shown to be increased in medical students in the study of anatomy, when this is provided in a clinical context ( Böckers *et al.*, 2014; Smith *et al.*, 2017), thereby making the content more relevant for them. Students see the ‘bigger picture’ and better understand why it is important that they have a firm grasp of the anatomical content.

Using evidence-based pedagogy on reflection and engagement, ‘dissection handover diaries’ were introduced to the first year of the clinical anatomy course in an undergraduate MBChB programme. These diaries were designed to aid students in reflecting on their understanding of anatomical concepts and on their ability to apply this knowledge to clinical scenarios. As noted by Cullinane and Barry (2023), student preference should be considered in anatomy curricula. Student engagement will be maintained when students perceive that the activity is beneficial to their learning and this engagement with the activity is important for it to have the desired effect. Therefore, a survey was developed to evaluate student perception of the diaries and to determine whether students considered completing the diaries useful to their learning.

## Methods

### Ethical statement

The study received ethical approval from the CAHID ethics committee of the School of Science and Engineering, University of Dundee (UOD_SSEREC_CAHID_STAFF_2023_04). Student participation in the survey was completely voluntary and completely anonymous. Students were asked to read a Participant Information Sheet and sign a consent form if they wished to participate in the survey. The signed consent form was kept separate from the completed study at all times and there was no identifying information collected as part of the survey.

### Dissection handover diaries

As part of the MBChB Year one Gastrointestinal clinical anatomy teaching sessions, student dissection teams were asked to complete a dissection handover diary at the end of each gross clinical anatomy dissection session. The diary comprised three short sections; ‘Description’ – here students were asked to provide their own description of the dissection session, including anything of anatomical or clinical note that was discovered; ‘Reflection’ – here students were asked to reflect on the session and note positive aspects, as well as any difficulties that arose, whether in the practical process or in the understanding of the underlying theoretical knowledge, and to think about what they could do as a team to learn from this; and ‘Clinical anatomy questions’ – here students were asked to answer a small number of clinical anatomy based questions that were related to the anatomy dissected that day, as well as being linked to assessed learning outcomes. The students were asked to complete this as a team and to have it signed off by a demonstrator prior to leaving the session. In this way staff could ensure that students had a solid grasp of the anatomy and its clinical relevance, as well as help rectify any misunderstandings before the students leave the dissecting laboratory. The next dissection group were then encouraged to read over the diary before starting their own dissection. The Year 1 students had previously undergone two blocks (Cardiovascular and Respiratory) during which they had participated in anatomical dissection but had not completed dissection handover diaries after each session.

### Survey

**Figure 1.**
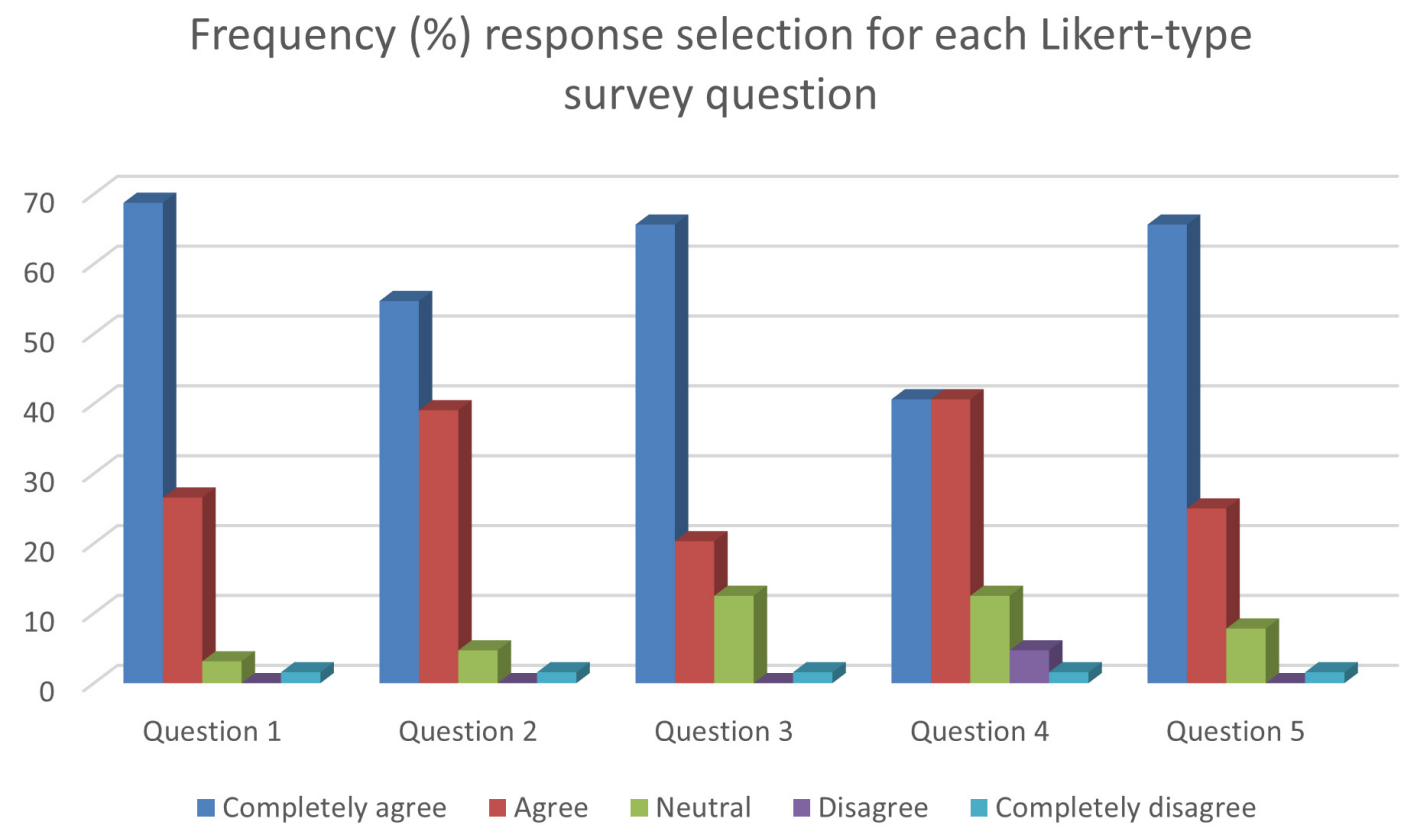
Chart showing the frequency (%) response selection for each Likert-type survey question.

Following the last dissection class of the block, students were asked to fill in a survey based on their perceptions of the diaries. This occurred over two class sessions on Monday 20 ^th^ March 2023. The survey was distributed in English and printed copies were made available for students to fill in anonymously before they left the dissection lab. The survey was designed to collect quantitative data in the form of five Likert-type questions, as well as collecting qualitative data in the form of a narrative constructive comments question, and was developed based on similar studies investigating student preference in dissection based activities ( Ali *et al.*, 2015; Asante *et al.*, 2021). The survey was not piloted prior to this study and no changes were made to the survey during the course of the study. The questions asked were designed to elicit student perceptions on the usefulness of the diaries, on their ability to encourage reflection, clinical application and student engagement in the sessions ( Figure 1). The students were asked to answer the questions based on a five point Likert-scale, with one being ‘Completely disagree’ and five being ‘Completely agree’. Students were also asked if they had any constructive comments to make about the diaries in a free-text response question ( McDougall, 2023).

### Analysis

The survey was completed by 64 students, from a total of 228 potential students – a response rate of 28%. The reliability of the survey was assessed using Cronbach’s alpha which gave a value of 0.88, indicating that the survey was a reliable instrument for assessing student perceptions of the diaries. According to Boone and Boone (2012), because Likert-type items exist on a scale where the measurement between each choice is not implicit - i.e. the choices for each Likert -type item can be on a higher or lower point scale, but there is not a measurable distance between those points – Likert-type items should be considered as ordinal data. As the data was treated as ordinal data, the mode was determined for each question rather than mean. The survey results are reported as frequency distributions.

Of the 64 respondents, 31 also completed the free-text response qualitative question of the survey. The qualitative free-text responses were collated and analysed for content using an inductive thematic analysis, with no preconceived expectations for the outcome. This involved the investigator reading through the comments individually, forming coding to represent patterns and themes before reviewing content again to ensure all data was assigned to the most appropriate theme.

## Results

The individual results of the five-point Likert-type questions section of the survey are shown in Table 1, as well as visually represented in Figure 1. The mode was returned as ‘5’ on the five-point scale, for each question item. This indicates that for each question ‘completely agree’ was the most frequently selected answer by participants ( McDougall, 2023).

**Table 1.** Number and percentage of participants selecting each response for each question.

Question		Completely agree		Agree		Neutral		Disagree		Completely disagree	
		N	%	N	%	N	%	N	%	N	%
1	Do you agree that the handover diaries are useful in allowing reflection on the practical session?	44	68.75	17	26.56	2	3.12	0	0	1	1.56
2	Do you agree that the handover diaries are useful for consolidating the anatomical theory relevant to the session?	35	54.69	25	39.06	3	4.69	0	0	1	1.56
3	Do you agree the handover diaries provide a clinical link to anatomy?	42	65.63	13	20.31	8	12.50	0	0	1	1.56
4	Do you agree the handover diaries are easy to complete?	26	40.63	26	40.63	8	12.50	3	4.69	1	1.56
5	Do you agree the handover diaries facilitate more student engagement with the practical session?	42	65.63	16	25.00	5	7.81	0	0	1	1.56

The completely agree and agree responses were combined, while the completely disagree and disagree responses were combined, to give an overall positive or negative rating for each question. Question 1 which asked students about the usefulness of the diaries for reflection returned a 95.3% positive rate. Question 2, which asked students about the usefulness of the diaries for consolidation of anatomical theory returned a 93.8% positive rate. Question 3 which asked students whether they perceived a clinical link to anatomy through the diaries returned a positive rate of 85.9%. Question 4, which asked whether the diaries were easy to complete, returned a positive rate of 81.2%. Question 5 which asked the students whether they perceived the diaries to have an effect student engagement returned a positive rate of 90.6%.

Analysis of the free text responses led to the identification of six main themes which were discussed in the student comments including reflection/consolidation, clinical anatomy, revision/assessment, diary approach, engagement and general usefulness. Some of the student comments related to more than one theme. Of the 31 comments, 14 either specifically mentioned, or were related to reflection and consolidation of material. Comments made by students related to this theme included: “I find the diaries very helpful when reflecting on what I've seen it also helps to know what the other group have done” (Participant ID (PID) 58, “Good to have reflection and relevant questions immediately after sessions to consolidate info while still fresh” (PID 38), and “Very helpful to summarise with my team, challenging questions sometimes but never too hard” (PID 16) ( McDougall, 2023). 11 of the responses specifically discussed, or related to the approach of the diaries. Comments on this theme included “Please continue to use them. They were very useful. Could add diagrams of specific/relative anatomical sites from that dissection” (PID 23), “I appreciate the separate sections (description, reflection, clinical appreciation (sic)) as it ties the session together well” (PID 64), and “More space for some questions - especially ones where drawing is required” (PID 55). Clinical anatomy was highlighted specifically in six of the comments made. Examples of the comments from this theme include “The questions at the end were good as they allowed me to think more about what we had been dissecting and link it to clinical information” (PID 1) and “Questions could have more clinical relevance - like enlarged pancreas/duodenal papillae question” (PID 38). There were four comments which related to the theme of revision/assessment, with example comments including “Thank you for introducing these as they have good questions and are a good starting point for revision” (PID 46) and “Multiple choice questions as well as written could be good” (PID 5). Three of the comments documented issues of student engagement, with examples including “I think they are very useful, however some in the group don't engage with it” (PID 28), and “The second section with clincial (sic) anatomy is quite difficult as it is additional info from whats (sic) in the book so if you haven't studies it yes its hard but 100% helps engagement and learning” (PID 8). Finally, three of the comments related to general usefulness of the diaries without linking this to any particular factor. Examples of the comments from this theme include “Not really, I thought they were quite useful” (PID 29) and “I thought they were very useful”(PID 32).

## Discussion

The aim of this study was to evaluate student perceptions of newly introduced dissection handover diaries in an undergraduate MBChB anatomy course, in the context of their usefulness to student learning. The results of this study, with the students providing a positive overall assessment of the diaries, are in broad agreement with studies investigating similar approaches to anatomical learning ( Abrams *et al.*, 2021; Lachman & Pawlina, 2006; O’Loughlin & Griffith, 2020).

As has been demonstrated, it is widely accepted that using reflective writing as a tool to help establish professional identity in medical students has positive outcomes ( Lachman & Pawlina, 2006; Mamede & Schmidt, 2004; Sandars, 2009; Wittich *et al.*, 2013). There appears to be a slow but steady increase in studies examining reflective writing specifically in anatomical education in the context of establishing a professional identity ( Alt-Epping *et al.*, 2014; Anstey *et al.*, 2014; Shiozawa *et al.*, 2020; Wittich *et al.*, 2013), however there remains a distinct paucity of information on reflective writing in anatomy in the context of improving anatomical knowledge ( Abrams *et al.*, 2021). By further understanding student perceptions of the diaries and their perceived usefulness to improving anatomical learning through reflecting, engaging and applying knowledge in a clinical context, this study contributes to the literature of using reflective writing approaches to improve anatomical knowledge and identifies the positive attitude with which it is received by students.

The students involved in the study had previously undertaken dissection sessions as part of other system blocks (respiratory and cardiovascular). The dissection sessions for all three blocks are highly similar in that many of the factors involved remain the same within and between blocks. This includes the setting in which the dissection takes place, staff support during the session, resources related to the session, including preparatory material, cadavers used, approach to dissection and length of time in sessions. The only factors which differed were the body system being dissected, in this case gastrointestinal and the use of the diaries. This meant that the students had a comparative aspect on which to base their perceptions of the diaries and whether they felt it impacted their anatomical learning. As an extension of this, one student commented as follows: “I really like them. I am a repeat student and have definitely found the diaries make a difference - knowing what the previous group has done is very helpful and having a proper debrief and discussion at the end of dissections has been very useful for consolidation” (PID 12). This, taken together with the overall positive results from the Likert-type questions, demonstrates that students do believe the diaries are useful and have helped them in their anatomical learning, when compared to similar sessions in which there were no dissection handover diaries used. This is an important aspect as previous studies, specifically related to anatomical education, have argued that ensuring student engagement in the activities of an anatomy curricula relies on students exhibiting a preference for the activity, and in doing so understanding the potential benefits to their learning experience ( Cullinane & Barry, 2023; Davis *et al.*, 2014; Phillips *et al.*, 2012).

While the results of this study are positive overall, students did provide constructive feedback on the format of the diaries, particularly in regard to providing more examples of clinical anatomy questions, providing more space for writing or drawing, and providing the answers to the clinical questions on their virtual learning environment. Many of these comments have been taken on board for the next iteration of the diaries. In the context of providing more examples of clinical anatomy questions, as has been discussed previously, studies have shown that medical students express a preference for learning anatomy when it is placed in a clinical context ( Böckers *et al.*, 2014; Smith *et al.*, 2017) – therefore it is not surprising that there is a request for more clinical anatomy questions. Each dissection diary ended with three clinically related questions, all of which related to the anatomy dissected during the current session. Due to time constraints within the session, the diaries were designed to be relatively quick and easy to fill in, therefore while giving providing more clinical anatomy based questions may be the preference for some students, this may have an impact on overall time and ease with which the diaries can be completed. However, it is noted that students would also prefer to have answers to the clinical questions provided on their virtual leaning environment rather than only in person with their demonstrators during the practical session. Model answers can be perceived as a form of feedback for students ( Huxham, 2007), providing them with a fast, informative and effective communication on whether they have grasped the concepts tackled in the sessions. Therefore, the intention is to provide students with model answers for the dissection diary clinical questions in the upcoming academic year.

There are limitations to the study including the fact that due to no identifying information being collected for any students, thus there was no way to drill down further into the data to establish whether there were trends in the survey results which correlated to differences in gender, age, or experience levels among students. This may be useful to know for future dissection diary revisions, in order to tailor the diaries as closely as possible to the needs and preferences of the majority of students. It would also be useful to consider whether the diaries did measurably improve anatomical learning in the context of assessed outcomes rather than purely evaluating student perceptions. This is a consideration for future studies, however for this initial study it was believed that evaluating student perceptions was an important first step in ensuring engagement with an activity that is designed to enhance educational experience.

## Conclusions

In conclusion, an evaluation of student perceptions of the newly introduced dissection handover diaries showed that students identified the diaries as being useful to their anatomical education by allowing them to reflect on the dissection session that had taken place, to consolidate their understanding and contextualise their learning with regard to clinical application. Future studies will determine whether this perception of usefulness correlates to a measurable increase in anatomical knowledge and understanding.

## Data availability

### Underlying data

Discovery: Student perceptions of handover diaries and reflective learning in an undergraduate MBChB anatomy course. https://doi.org/10.15132/10000241. ( McDougall, 2023).

The project contains the following underlying data:

- Perceptions_of_Dissection_Diaries_Questionnare_Data_Set_-_Quantitative.xlsx (Quantitative (Likert-type survey results) data set supporting the results of a survey developed to assess student perceptions of a dissection diary and reflective learning).
- Perceptions_of_Dissection_Diaries_Questionnare_Data_Set_-_Qualitative_Comments.xlsx (Qualitative (student open comments) data sets supporting the results of a survey developed to assess student perceptions of a dissection diary and reflective learning.)

### Extended data

Discovery: Student perceptions of handover diaries and reflective learning in an undergraduate MBChB anatomy course. https://doi.org/10.15132/10000241. ( McDougall, 2023).

This project contains the following extended data:

- Survey_-_Handover_diaries_for_MBChB_Anatomy_classes_SMcD_150223_v1.docx (Blank copy of survey used in the above named project).

### Reporting guidelines

Discovery: SRQR checklist for: Student perceptions of handover diaries and reflective learning in an undergraduate MBChB anatomy course. https://doi.org/10.15132/10000241. ( McDougall, 2023).

Data are available under the terms of the Creative Commons Attribution 4.0 International license (CC-BY 4.0).
