# Supplementary material for: Student perceptions of handover diaries and reflective learning in an undergraduate MBChB anatomy course
Source: MedEdPublish (2016). 2023 Dec 5;13:305. [Version 1] doi: 10.12688/mep.19946.1 (PMC11384193; doi:10.12688/mep.19946.1)
Supplement: Peer review history

# Reviewer report: Reviewer response for version 1

**DOI:** 10.21956/mep.21363.r38395

**Published:** 2024-09-09

**Authors:** Jo Bishop

**Affiliations:** 1 Bond University, Queensland, Australia

Student perceptions of handover diaries and reflective learning in an undergraduate MBChB anatomy course. [version 1; peer review: awaiting peer review]

 The authors consideration of reflective practice to support engagement of students is a great initiative as the health professional education community consider programmatic assessment and integration of competency-based assessment through experiential learning to enhance professional identity and ‘expert learners. The authors have reviewed key areas of the literature to validate their work.

- The authors should consider using the generic term of medical degree rather than MBChB for international relevance in the title, abstract and in introductory commentary (and keywords). There should be consideration of the length of the program (five years?).
- The dissection handover diaries were explained – it would be opportunistic if students had previously had opportunities to reflect in the program to date (is this known)– was there a session to clarify the task? Although it was a team activity that was signed off by a demonstrator – how reassured are the authors of full engagement by the team/all students.
- In what modality was the diary, online/ ipad, paper (as it takes place in the dissection room)– could a student complete individually and retrospective as part of their own record? I note in the discussion that the ‘model answers’ will be made available to students on the virtual learning environment as part of feedback opportunity.
- How many dissection sessions were there? Not sure if the date is relevant to this publication (survey section)

The authors state reliability with the number of responses – the results section is clearly written with quantitative and qualitative data analysed.

 The discussion is well written and considers strengths and limitations and I would support further consideration and how this supports engagement programmatically across the cohorts learning. Reviewing knowledge and understanding retrospectively with cohorts who have used diaries and those who haven’t would be beneficial.

Have any limitations of the research been acknowledged?

Yes

Is the work clearly and accurately presented and does it cite the current literature?

Yes

If applicable, is the statistical analysis and its interpretation appropriate?

Yes

Are all the source data underlying the results available to ensure full reproducibility?

Yes

Is the study design appropriate and is the work technically sound?

Yes

Are the conclusions drawn adequately supported by the results?

Yes

Are sufficient details of methods and analysis provided to allow replication by others?

Yes

Reviewer Expertise:

I am an anatomist and head of curriculum at a med school and have 20 years experience

I confirm that I have read this submission and believe that I have an appropriate level of expertise to confirm that it is of an acceptable scientific standard.

# Reviewer report: Reviewer response for version 1

**DOI:** 10.21956/mep.21363.r35611

**Published:** 2024-08-13

**Authors:** Yelda Pinar

**Affiliations:** 1 Anatomi Anabilim Dalı, Ege University, İzmir, Turkey

The article offers a new and different method in anatomy education. It was an interesting study in this respect. While reading this article, I came across expressions such as reflective writing and handover diaries. I did some research on this subject. I am also an anatomist, but I am unfamiliar with these expressions, especially diaries.

 Of course, it is sad that the number of students is high and the number of participants is low. However, the author also stated this at the end of the discussion, saying that a new study should be done. Only the method needs a little more explanation in the Dissection handover diaries section; because he is talking about a new learning method here, it should be clearer and more understandable.

 An example of the handover diaries that students are asked to write can be included in the article as a picture.

Have any limitations of the research been acknowledged?

No

Is the work clearly and accurately presented and does it cite the current literature?

Yes

If applicable, is the statistical analysis and its interpretation appropriate?

Yes

Are all the source data underlying the results available to ensure full reproducibility?

Yes

Is the study design appropriate and is the work technically sound?

Yes

Are the conclusions drawn adequately supported by the results?

Yes

Are sufficient details of methods and analysis provided to allow replication by others?

Partly

Reviewer Expertise:

head and neck anatomy, clinical anatomy, anatomy education, esthetic anatomy

I confirm that I have read this submission and believe that I have an appropriate level of expertise to confirm that it is of an acceptable scientific standard.
